# Hydroethanolic Extract from *Bridelia atroviridis* Müll. Arg. Bark Improves Haematological and Biochemical Parameters in Nicotinamide-/Streptozotocin-Induced Diabetic Rats

**DOI:** 10.1155/2020/3160834

**Published:** 2020-11-25

**Authors:** Clarice Noussi Djouwoug, Florence Tsofack Ngueguim, Raceline Kamkumo Gounoue, Clémence Donfack Gouni, Antoine Kavaye Kandeda, Jean Philippe Djientcheu, Rodrigue Fifen, Desire Paul Djomeni Dzeufiet, Silvère Ngouela, Norbert Sewald, Bruno Ndjakou Lenta, Theophile Dimo

**Affiliations:** ^1^Department of Animal Biology and Physiology, Faculty of Science, University of Yaoundé I, P.O. Box 812, Yaoundé, Cameroon; ^2^Department of Organic Chemistry, Faculty of Science, University of Yaoundé I, P.O. Box 812, Yaoundé, Cameroon; ^3^Department of Chemistry Organic and Biorganic Chemistry, University of Bielefeld, P.O. BOX 100131, D-33501, Bielefeld, Germany; ^4^Department of Chemistry, Higher Teacher Training College, University of Yaoundé 1, P.O. Box 47, Yaoundé, Cameroon

## Abstract

*Bridelia atroviridis Müll. Arg. (B. atroviridis*) is a plant used in Cameroonian traditional medicine to manage diabetes. The effects of hydroethanolic barks extract from *B. atroviridis* were evaluated on diabetes disorders including hematology, inflammatory, and oxidative stress parameters. The in vitro antioxidant capacity of the hydroethanolic bark extract (70 : 30) was evaluated. Nicotinamide-/streptozotocin-induced diabetic rats were daily treated with the *B. atroviridis* extract for fifteen days. Glycemia were evaluated every 5 days, insulin sensibility test was performed, and haematological, inflammatory, and oxidative stress parameters were analysed. Histomorphometry of the pancreas was realized. The extract was able to scavenge free radicals in vitro and decrease significantly the blood glucose levels. The treatment resulted in a significant alleviation of insulin resistance, anemia, leukocytopenia, and thrombocytopenia observed in untreated diabetic rats. The extract significantly decreased proinflammatory cytokines TNF-*α*, IL-1*β*, and IL-10. The rate of reduced glutathione was increased in the pancreas, whereas the catalase activity and nitrite concentration were decreased. Diabetic control showed a reduced size of Langerhans islet, whereas the size of islets was large in treated groups. The hydroethanolic extract of *B. atroviridis* was able to improve glycemia and alleviate haematological and inflammatory parameters disorders observed in diabetic conditions, probably due to its antidiabetic, anti-inflammatory, and antioxidant capacities.

## 1. Introduction

Diabetes is a metabolic disorder characterized by chronic hyperglycemia resulting from deficiency of secretion (type I diabetes) and/or insulin action (type II diabetes) [1]. In 2017, the International Diabetes Federation (IDF) estimated about 425 million adults with diabetes all over the world with 16 million in Africa, the number of diabetic patients is still growing worldwide, and more than 629 million could be suffering in 2045 [2]. This may be due to some disadvantages provided by the most available treatment, including drug resistance (reduction of efficiency), side effects, and even toxicity [3]. Morbidity and mortality could be due to the complications often occurred in this disease [4]. Microvascular changes are the main consequence of chronic hyperglycemia and lead to blindness, amputations, kidney disease, anemia, cardiovascular attack, brain complications, impairment in immunological response, and gastrointestinal system [5, 6]. In diabetic state, lipotoxicity and glucotoxicity generate oxidative stress and exacerbate inflammatory response [7].

Chronic hyperglycemia has a direct relationship with the development of an inflammatory condition marked by the increased expression of proinflammatory cytokines such as IL-1*β*, IL-6, TNF-*α*, and NF*κ*B [8]. Evidence of these immunological changes involves variation in the levels of cytokines and chemokines, changes in the numbers and activation states of various leukocyte populations, apoptosis, and fibrosis. These disturbances are observed in the adipose tissue, liver, pancreatic islets, kidney, and vasculature [7].

The cumulative results of these transformations resulted from excess generation of reactive oxygen species (ROS) mediated by chronic hyperglycemia [9]. The development of complications related with diabetes takes place with ROS production, mainly O_2_^−^ which induces cell dysfunction and oxidative lesion by protein denaturation pathway, lipid peroxidation, and damage to mitochondrial DNA [9, 10].

Recent studies showed that the majority of plasma antioxidants are depleted in diabetic state [11]. Therefore, future treatment of type 2 diabetes and its complications may include hypoglycemic, anti-inflammatory, and antioxidant drugs [7, 11]. Traditional medicine systems generally assume that synergy effects of all ingredients of the plants will bring about maximum therapeutic [12]. In Cameroon, particularly in the centre region (Mbalmayo), *Bridelia atroviridis Müll. Arg.* (Euphorbiaceae) is used by local population to manage various ailments including diabetes, malaria, and venereal diseases. *B. atroviridis* methanolic leaves and stem bark extracts possess antifungal activity, and the decoction from its bark is used as purgative, diuretic, and aphrodisiac remedy [13]. Although ethnobotanical surveys of certain medicinal plants used in Cameroon do not indicate the traditional use of *B. atroviridis* on diabetes [14], some plants of the genus Bridelia have been cited as antidiabetic [13]. Meanwhile, the antidiabetic activity of *Bridellia ferruginea* [15], *Bridelia micrantha* [16], *Bridelia ndellensis* Beille [17], *Bridellia grandis* [18], and *Bridellia retusa* [19] has been proven. It was also reported that barks of all *Bridellia* Spp have similar medicinal uses in West Africa [20] motivating the investigation of the antidiabetic activity of *Bridelia* hydroethanolic extract. Hereby, we investigated the possible bioactivities of *B. atroviridis* hydroethanolic extract against some pathophysiological effects on nicotinamide-/streptozotocin-induced diabetes in rats.

## 2. Materials and Methods

### 2.1. Plant Material and Extract Preparation

*Bridelia atroviridis* barks were harvested at Mbalmayo in December 2018. The plant was authenticated by Mr. Ngansop Eric, a botanist at the National Herbarium, Yaoundé (Cameroon), in comparison with the specimen voucher N35241/HNC Cam. The barks were air-dried in the shade and pulverized into powder. A kilogram of powder from *B. atroviridis* was soaked in 5 L of hydroethanolic solvent mixture (30% water and 70% ethanol; v/v) for 72 hours with regular agitation. Then, the mixture was filtered with Whatman paper No. 3, and the filtrate was evaporated to dryness using a rotatory evaporator (Buchi Rota vapor, Switzerland) at a temperature of 45°C. The residual water was removed by ventilation under a hood (Burdinola ST 1800) giving a powdered crude extract.

### 2.2. Phytochemical Analysis

The extract was screened for detection of different chemical families according to the standardized methods described by Odebiyi and Sofowora [21] and also by Gul et al. [22]. Briefly, phenolic compounds were detected using the ferrocyanide reaction; triterpenes and sterols were revealed by their reactivity with anhydrous acetate and sulphuric acid. Alkaloids were detected using Mayer's reagent, whereas the presence of saponins was revealed based on their foaming property. Tannins and flavonoids were revealed using ferric chloride and hydrochloric acid, respectively. Anthraquinones were detected in extract by the chloroform/petroleum system.

### 2.3. In Vitro Antioxidant Activities of *B. atroviridis*

#### 2.3.1. DPPH Test

This test was performed as described by Aadil and collaborators [23]. The radical scavenging activities of the plant extract were evaluated spectrophotometrically using the 1,1-diphenyl-2-picrylhydrazyl (DPPH) free radical. When DPPH reacts with an antioxidant compound, which can donate hydrogen, it is reduced. The color change was measured at wave length, 517 nm under UV/Visible light spectrophotometer. 50 *μ*L of the diluted extract (1000 *μ*g/mL in ethanol) were mixed with 150 *μ*L of 1,1-diphenyl-2-picrylhydrazyl (DPPH) ethanol solution and then diluted twofold serially resulting to a final extract concentrations range from 250 to 1.9531 *μ*g/mL. After 1 hour of incubation in the dark at room temperature, the optical densities were measured. Ascorbic acid (Vitamin C) was used as control. The radical scavenging activity fifty (RSA_50_, in %) was calculated [23].

#### 2.3.2. ABTS Assay

The radical scavenging activities of crude extract were evaluated spectrophotometrically using the 2,2′-azino-bis (3-ethylbenzothiazoline-6-sulphonic) acid (ABTS) free radical as described by Aadil and collaborators [23]. The extract (1000 *μ*g/mL) was three-fold serially diluted with ethanol. 50 *μ*L of the diluted extract was mixed with 150 *μ*L of 2,2′-azino-bis (3-ethylbenzothiazoline-6-sulphonic) acid (ABTS) solution, giving a final extract concentration range of 250–1.9531 *μ*g/mL. Ascorbic acid (Vitamin C) was used as control and after 1 h incubation in the dark at room temperature; the optical densities were measured at 734 nm. The radical scavenging activity fifty (RSA_50_, in %) was calculated [23].

#### 2.3.3. Nitric Oxide Radical Scavenging Activity

Nitric oxide generated from sodium nitroprusside in aqueous solution interacts with oxygen to produce nitrite ions, measurable through the Griess reaction. The test was preformed according to Hossain and collaborators [24] with slight modifications. Briefly, in 75 *μ*L of sodium nitroprusside, 50 *μ*L of extract was added at different concentrations leading to a final extract concentration from 250 to 1.9531 *μ*g/mL. The mixture was then incubated at room temperature for 2 hours. The blank was prepared by replacing extract with ethanol. At the end of the incubation time, 1.25 mL of Griess reagent (1% sulfanilamide in 5% phosphoric acid and 0.1% N-1-napthylethylenediamine dihydrochloride in water) were added, and the absorbance was recorded after 5 min in the dark at 540 nm. Inhibition percentages of the nitrite oxide generated were measured by comparing the absorbance values of test samples versus control. Ascorbic acid was used as a positive standard control in the study.

#### 2.3.4. Ferric-Reducing Antioxidant Power (FRAP) Assay

The ferric reducing power was determined by the Fe^3+^-Fe^2+^ transformation in the presence of the extract [25]. The Fe^2+^ was monitored by measuring the formation of orthophenanthroline at 505 nm. The extract (1000 *μ*g/mL) was twofold serially diluted with ethanol. 50 *μ*L of the diluted extract was mixed with 50 *μ*L of ferric chloride solution. After 15 min of incubation in the dark at room temperature, 50 *μ*L of orthophenanthroline was added to obtain final extract concentrations ranging from 250 to 1.9531 *μ*g/mL (250, 125, 62.5, 31.25, 15.625, 7.8125, 3.9062, and 1.9531 *μ*g/mL). Hydroxylamine (NH_2_OH) was used as positive control, and the optical densities were measured at 505 nm; each assay was done in triplicate. The Fe^3+^-reducing capacity (RC in %) was calculated, the reduction percentages were plotted against the logarithmic values of concentration of test samples, and a linear regression curve was established in order to calculate the inhibitory concentration 50 (IC_50_), which is the concentration of sample necessary to reduce 50% the total free Fe^3+^ in Fe^2+^ radical.

### 2.4. Animals

Rats weighing 220 ± 20 g were used in the present study. They were obtained from the animal house of the Faculty of Science, University of Yaoundé I (Cameroon). Animals were submitted to the standard diet established in this laboratory and received water *ad libitum*. All the procedures in the present study followed the principles of laboratory animal use and care of the “European community guidelines (EEC Directive 2010/63/EEC) and were approved by the “Animal Ethical committee” of the Faculty of Science, University of Yaoundé I.

### 2.5. Induction of Type 2 Diabetes and Experimental Design

Type II diabetes was induced by the intraperitoneal injection of 110 mg/kg of nicotinamide 15 min prior intravenous (penile vein) injection of 55 mg/kg streptozotocin in 0.9% of sodium chloride solution [26]. Diabetes was allowed to develop and to stabilize over a period of 2 weeks [27], animals with glycemia between 126 and 300 mg/dL were considered as diabetic, and then they were subjected to the insulin resistance test. Diabetic animals were divided into 5 groups of 5 animals each and treated as follows: one group treated with distilled water (10 mL/kg) named diabetic control, another group consisted to metformin 200 mg/kg treated with metformin (200 mg/kg), and three test groups that received the hydroethanolic bark extract of *B. atroviridis* at the respective dose of 50, 100, and 200 mg/kg. To these different groups, one normal control group was made up of healthy rats, and receiving distilled water was added. Animals were orally treated for 15 days during which fasting blood glucose levels was recorded every five days from the tail's blood drop, using a glucometer (Accucheck Active). At the end of the treatment, animals were sacrificed under anaesthesia using ketamin (30 mg/kg) and diazepam (10 mg/kg). Blood sample of each animal was collected into EDTA tubes and dry tubes. The pancreas was harvested for oxidative stress and histomorphometry analysis.

### 2.6. Insulin Tolerance Test (ITT)

At the beginning and the end of the treatment, the insulin tolerance test was performed to evaluate the insulin sensitivity. A dose of 0.15 IU/kg of semislow insulin was injected subcutaneously to diabetic rats prior to a 12 h nonhydrated fasting as described by Patarrão et al. [28] with slight modifications. The glycemia was measured after insulin injection (*t* = 0 min) and then successively after 15, 30, and 60 min insulin injection. The slope of the linear decline in plasma glucose (*K*_ITT_) was calculated according to the following formula: *K*_ITT_ = (0.693/*T*_1/2_) × 100 [28, 29]. *T*_1/2_ represents the half life of plasmatic glucose.

### 2.7. Haematological Analysis

EDTA blood sample collected from each animal was used to performed different blood parameters using the Sysmex hematometer 300 (Sysmex300, Germany).

### 2.8. Serum Inflammatory Parameter Analysis

Blood sample into dry tubes was centrifuged, and supernatant serum was analysed for measure of some inflammatory parameters such as TNF-*α*, IL-1*β*, Il-6, and IL-10 using Quantikine Elisa kits (Germany).

### 2.9. Oxidative Stress Parameter Analysis and Histopathological Analysis of the Pancreas

The pancreas is the central organ involved in the pathogenesis of both type I and type II diabetes. To evaluate the oxidative stress balance in this organ, a part of the organ was homogenate in Tris HCL buffer (10%) and some markers of oxidative stress such as the rate of reduced glutathione and nitrites, and the activity of catalase enzyme were evaluated. Histomorphometry study of the remaining part of pancreas was performed after haematoxylin/eosin stain. The area of pancreatic islet was measured using software of area measurement (Image J. version 1.3).

### 2.10. Statistical Analysis

Results were expressed as mean ± S.E.M. Statistical differences between control and treated groups were highlighted using one-way analysis of variance (ANOVA) followed by Turkey's multiple comparison test, and Student *t* test was used to compare in vitro anti-oxidant test using Graphpad Prism 7 software. *P* values less than 0.05 were considered as significant.

## 3. Results

### 3.1. Phytochemical Qualitative Content

Qualitative analysis of the plant extract revealed the presence of alkaloids, flavonoids, phenols, tannins, triterpenes, steroids, saponins, and anthocyanins, whereas diterpenes, anthraquinone, glucosides, and coumarins were absent.

### 3.2. In Vitro Scavenging and Antioxidant Activities of *B. atroviridis*

Table 1 indicates the radical scavenging activities of *B. atroviridis* on DPPH and ABTS. The results show that *B. atroviridis* expresses better antioxidant activities compared to the control. The plant extract showed greater DPPH scavenging than vitamin C and its capacity to catch ABTS is closed to the ascorbic acid. By contrary, the extract exhibited poor NO scavenging effect than the known standard. Concerning the capacity of *B. atroviridis* to prevent the reduction of Fe^3+^ to Fe^2+^, it appears that the plant extract was able to inhibit the transformation of Fe^3+^ to Fe ^2+^ but lesser than hydroxylamine used as the standard.

### 3.3. Insulin Sensitivity Test

**Figure 1:**
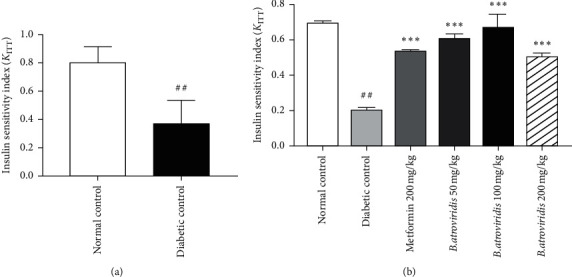
Insulin index before treatment (a) and the effects of *B. atroviridis* on insulin index (b) after 2 weeks of treatment. Each bar represents the mean ± SEM (*n* = 5); ##*P* < 0.01 and ###*P* < 0.001 indicate significant difference between diabetic and normal control and *∗∗∗P* < 0.001 indicates significant difference between diabetic control and treated groups.

Fourteen days after the diabetes induction, insulin resistance was evaluated prior the beginning of the treatment. The administration of insulin to nicotinamide-/streptozotocin-induced diabetic rats failed to decrease the blood glucose levels (data not shown).  Figure 1 shows the level of insulin index (*K*_iTT_) before and after the treatment with the plant extract. Insulin injection provoked throughout the experimental period a drop of *K*_iTT_ by 53.90% (Figure 1(a)) as compared to normal rats. However, cumulative administration of the plant extract induced an improvement in insulin sensitivity characterized by a rise of *K*_iTT_. The *K*_iTT_ value of diabetic rats remained significantly low at the end of the experimental period compared to normal rats (71.65%, *P* < 0.001). The extract induced a rise in *K*_iTT_ by 67.44%, 64.50%, and 60.84% at the respective doses of 50 mg/kg, 100 mg/kg, and 200 mg/kg (Figure 1(b)).

### 3.4. Effect of Hydroethanolic Bark Extract of *B. atroviridis* on Body Weight Variation

**Figure 2:**
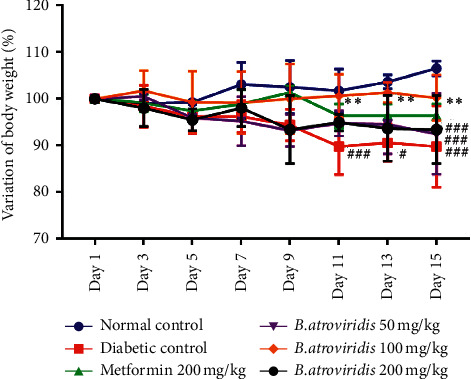
Effects of *B. atroviridis* extract on body weight variation. Each point represents mean ± SEM (*n* = 5). #*P* < 0.05 and ###*P* < 0.001: significant difference compared to normal control. *∗P* < 0.05 and *∗∗P* < 0.01: significant difference related to diabetic control. Normal control = healthy animals treated with distilled water (10 mL/kg). Diabetic control = nicotinamide-/streptozotocin-induced diabetic rats treated with distilled water (10 mL/kg). Metformin 200 mg/kg = nicotinamide-/streptozotocin-induced diabetic rats treated with metformin at the dose of 200 mg/kg. *B. atroviridis* 50 mg/kg, 100 mg/kg, and 200 mg/kg = nicotinamide-/streptozotocin-induced diabetic rats treated with *B. atroviridis* extract at the doses of 50 m/kg, 100 mg/kg, and 200 mg/kg, respectively.

Figure 2 presents the effects of *B. atroviridis* extract in nicotinamide-/streptozotocin-induced diabetic rats. The evolution of the body weight in the diabetic control group was lower compared to normal control. The administration of the plant extract for 15 days significantly improved the body weight gain compared to the diabetic control group only at the dose of 100 mg/kg from day 9 to day 15. The plant extract at the doses of 50 and 200 mg/kg did not significantly prevent the loss of body weight up to the end of the experiment. In contrary, the evolution of the body weight of animals in these groups, as in negative control group, was significantly low compared to the normal control.

### 3.5. Effects of Cumulative Administration of *B. atroviridis* Hydroethanolic Extract on Blood Glucose

**Figure 3:**
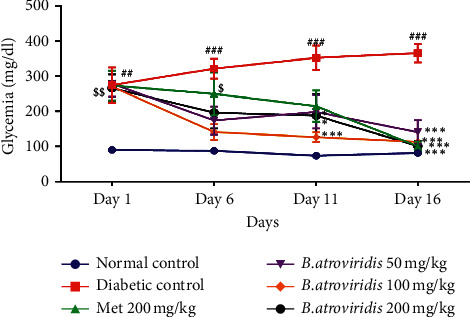
Effects of *B. atroviridis* on the evolution of glycemia. Each point represents mean ± ESM, *n* = 5. *∗P* < 0.05, *∗∗P* < 0.01, and *∗∗∗P* < 0.001 indicate significant difference between diabetic control and treat groups at a given day. ##*P* < 0.01 and ###*P* < 0.001 indicate significant difference between normal control and diabetic control and at a given day. $*P* < 0.01 and $$$*P* < 0.001 indicate significant difference between normal control and treated groups at a given day.

The injection of nicotinamide prior to streptozotocin administration induced hyperglycemia by 66.98%. The glycemia remained high by 76.36% at the end of the treatment compared to normal control rats. The single daily administration of *B. atroviridis* for 2 weeks caused a significant decrease (*P* < 0.001) in blood glucose by 59.24% (50 mg/kg), 67.05% (100 mg/kg), and 70.52% (200 mg/kg) compared to diabetic control (Figure 3). Compared to their initial value (day 1), it was observed a significant decrease in blood glucose levels by 47.97%, 57.93%, and 62.22%, respectively, at the dose of 50, 100, and 200 mg/kg. The decrease was gradual and significant from day 11. The plant extract at the dose of 200 mg/kg brought back the glycemia towards the normal value. Metformin administration at the dose of 200 mg/kg induced the decrease of blood glucose level by 70.52%.

### 3.6. Effects of Hydroethanolic Bark Extract of *B. atroviridis* in Some Haematological Parameters in Nicotinamide-/STZ-Induced Diabetes Rats

The induction of diabetes provoked anemia characterized by a significant drop of erythrocytes (RBC) count (31.85%, *P* < 0.01), hematocrit (HCT) (13.83%, *P* < 0.01), mean concentration of haemoglobin (MCH) by 38.26% (*P* < 0.01), mean corpuscular haemoglobin concentration (MCHC) (20.44%), and increase of mean corpuscular volume (MCV) (20.87%, *P* < 0.05) in the diabetic control group compared to normal control. In addition, the level of white blood cells increased (55.67%, *P* < 0.01), while the lymphocyte percentage decreased (24.96%, *P* < 0.01) (Table 2). The rate of platelets was also significantly decreased in the diabetic control group (123.65%, *P* < 0.001) compared to the normal control. Oral administration of the plant extract induced a significant increase (at least at *P* < 0.01) of RBC, HCT, lymphocytes, and platelet rate.

### 3.7. Effect of Hydroethanolic Extract of *B. atroviridis* on Some Inflammatory Parameters

**Figure 4:**
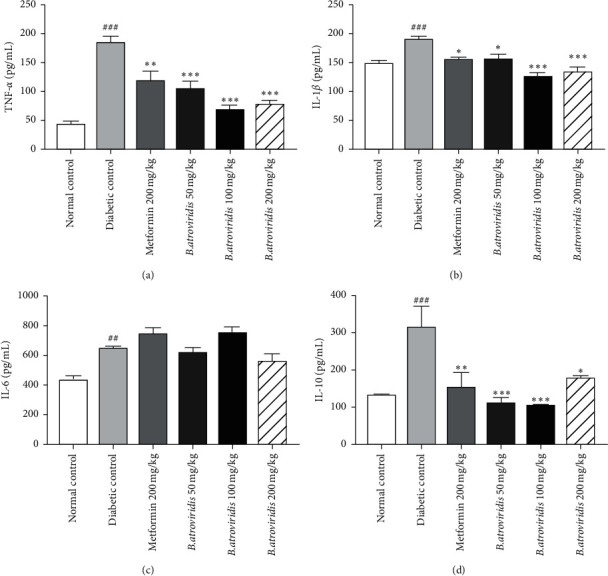
Effects of *B. atroviridis* on TNF-*α* (a), IL-1*β* (b), IL-6 (c), and IL-10 (d) concentrations in nicotinamide-/streptozotocin-induced-diabetic rats. Each bar represents the mean ± SEM (*n* = 5). #*P* < 0.05, ##*P* < 0.01, and ###*P* < 0.001 indicate significant difference between diabetic and normal control and *∗P* <  0.05, *∗P* < 0.01, and *∗∗∗P* < 0.001 indicate significant difference between diabetic control and treated groups. Normal control = healthy animals treated with distilled water (10 mL/kg). Diabetic control = nicotinamide-/streptozotocin-induced diabetic rats treated with distilled water (10 mL/kg). Metformin 200 mg/kg = nicotinamide-/streptozotocin-induced diabetic rats treated with metformin at the dose of 200 mg/kg. *B. atroviridis* 50 mg/kg, 100 mg/kg, and 200 mg/kg = nicotinamide-/streptozotocin-induced diabetic rats treated with *B. atroviridis* extract at the doses of 50 m/kg, 100 mg/kg, and 200 mg/kg, respectively. TNF-*α* = tumor necrosis factor alpha, IL-1*β* = interleukin 1 beta, IL-6 = interleukin 6, and IL-10 = interleukin 10.

The administration of streptozotocin-/nicotinamide-induced diabetes is responsible of an increase rate of proinflammatory cytokines such as TNF-*α* (Figure 4(a)), IL-1*β* (Figure 4(b)), and IL-6 (Figure 4(c)) by 76.81%, 22.27%, and 32.04, respectively, while the increase rate of the anti-inflammatory factor IL-10 was 58.41% (Figure 4(d)) as compared to normal control. The treatment with hydroethanolic extract of *B. atroviridis* induced a significant decrease (*P* < 0.001) of TNF-*α* by 43.78%, 62.92%, and 58.38%, respectively, at the doses of 50, 100, and 200 mg/kg. The extract also showed a significant decrease of IL-1*β* by 17.62% (50 mg/kg), 33.68% (100 mg/kg), and 29.53% (200 mg/kg) after 15 days of treatment. The IL-10 concentration decreased by 65.08% (*P* < 0.001), 67.30% (*P* < 0.001), and 44.44% (*P* < 0.05) at the respective dose of 50, 100, and 200 mg/kg. However, the plant extract failed to reduce (*P* > 0.05) IL-6 concentration whatever the dose is.

### 3.8. Effect of *B. atroviridis* Hydroethanolic Extract on Some Oxidative Stress Biomarkers in Pancreatic Tissue

**Figure 5:**
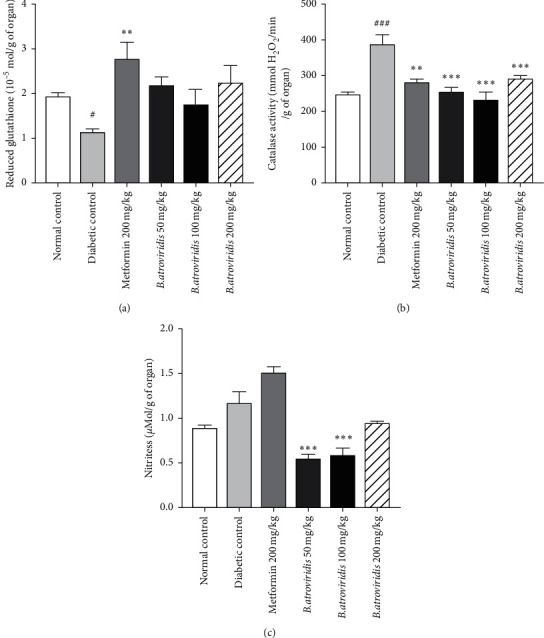
Effects of *B. atroviridis* on the rate of reduced glutathione (a), catalase activity (b), and rate of nitrites (c) in the pancreas. Bars represent mean ± SEM (*n* = 5). ##*P* < 0.01 and ###*P* < 0.001: significant difference compared to normal control. *∗P* < 0.05, *∗∗P* < 0.01, and *∗∗∗P* < 0.001: significant difference related to diabetic control. Normal control = healthy animals treated with distilled water (10 mL/kg). Diabetic control = nicotinamide-/streptozotocin-induced diabetic rats treated with distilled water (10 mL/kg). Metformin 200 mg/kg = nicotinamide-/streptozotocin-induced diabetic rats treated with metformin at the dose of 200 mg/kg. *B. atroviridis* 50 mg/kg, 100 mg/kg, and 200 mg/kg = nicotinamide-/streptozotocin-induced diabetic rats treated with *B. atroviridis* extract at the doses of 50 m/kg, 100 mg/kg, and 200 mg/kg, respectively.

Figure 5 expresses the effects of *B. atroviridis* on some oxidative stress parameters. The concentration of reduced glutathione significantly decreased (41.45%, *P* < 0.05) in diabetic rats, whereas the catalase activity significantly increased (36.34%, *P* < 0.001) compared to normal control. The administration of the plant extract for 15 days significantly decreased the catalase activity by 34.02% (50 mg/kg), 39.95% (100 mg/kg), and 25% (200 mg/kg) in comparison with the diabetic control. The concentration of nitrites in the diabetic control group was higher than that of normal control (23.76%), and the plant extract significantly reversed this concentration by 53.68% and 50.08% (*P* < 0.001) at the doses of 50 and 100 mg/kg, respectively, comparing to diabetic control rats.

### 3.9. Effects of *B. atroviridis* on Microarchitecture of Pancreas

**Figure 6:**
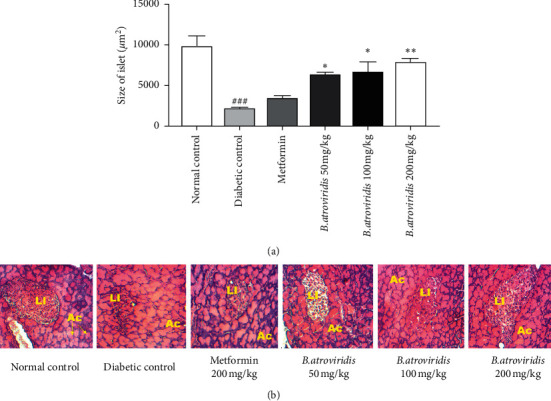
Effects of *B. atroviridis* on the size of Langerhans islets (a) and the microarchitecture of the pancreas in diabetic rats (b). Each bar represents mean ± SEM, *n* (number of Langerhans islets observed per animal) = 10; ##*P* < 0.001 indicates significant difference between diabetic and normal control *∗P* < 0.05 and *∗∗P* < 0.01 indicate significant difference between diabetic control and treated groups. LI: Langerhans islet, Ac: Acini. Normal control = healthy animals treated with distilled water (10 mL/kg). Diabetic control = nicotinamide-/streptozotocin-induced diabetic rats treated with distilled water (10 mL/kg). Metformin 200 mg/kg = nicotinamide-/streptozotocin-induced diabetic rats treated with metformin at the dose of 200 mg/kg. *B. atroviridis* 50 mg/kg, 100 mg/kg, and 200 mg/kg = nicotinamide-/streptozotocin-induced diabetic rats treated with *B. atroviridis* extract at the dose of 50 m/kg, 100 mg/kg, and 200 mg/kg, respectively.

Figure 6 describes the microarchitecture of the pancreas in the experimental animals. In diabetic control group, a significant reduction (78.08%, *P* < 0.001) in the Langerhans islets size was observed compared to normal control as shown in (a). The administration of the plant extract significantly increased the Langerhans islet size with dose-dependant effect at 50 mg/kg (65.80%, *P* < 0.05), 100 mg/kg (67.75%, *P* < 0.05), and 200 mg/kg (72.3% *P* < 0.01).

## 4. Discussion

*B. atroviridis* is largely used in Africa for many ailments [13], but its effect on some pathophysiological aspect in diabetic state was not scientifically studied up to now yet. In this study, nicotinamide-/streptozotocin-induced type II diabetes was characterized by a hyperglycemia, insulin resistance, anemia, leukocytosis, thrombocytopenia, inflammation, and structural damage of the pancreas. The administration of hydroethanolic extract of *B. atroviridis* bark for 15 days to diabetic rats efficiently corrected the blood glucose level with the marked effect at the dose of 200 mg/kg, effective as the reference drug metformin (200 mg/kg) at the end of the experimental period. The hypoglycemic effect observed could be due to the reduction of insulin resistance noted in treated rats. It is well known that metformin is equally acted by increasing the insulin sensitivity [30]. The plant extract may also act by stimulating the secretion of insulin and/or by increasing the insulin sensitivity [31]. These actions have been attested in the present study by the rise in insulin sensitivity index observed at the end of the treatment. Antihyperglycemic effect may be attributed to the presence of substances as flavonoids, alkaloids, saponin, tannins, and triterpenes contained in the extract. In addition, the determination of phytochemicals of hydroethanolic extract of *B. artroviridis* carried out by our research team revealed the presence of compounds such as myricetin and corilagin [32]. Corilagin acts by enhancing peripheral glucose utilization and stimulating pancreatic *β* cells to produce insulin [33] while myricetin enhances intracellular protein activity, encouraging glucose uptake consequently reduces insulin resistance [34, 35]. These different compounds might react synergically to contribute to the observed hypoglycemic effect of the plant extract. Although the highest dose of the extract (200 mg/kg) expressed the best activity, considering the hypoglycemic effect by the end of the treatment, the dose of 100 mg/kg showed the most interesting effect since it presents progressive significant hypoglycemia activity from day 10 compared to the other treated groups.

Permanent hyperglycemia in diabetes condition leads to increase expression of inflammatory markers like IL-6 and TNF-*α* [36] as observed in this study. These cytokines are produced by immune and nonimmune cells when challenged by various environmental or inflammatory insults. Chronic exposure to proinflammatory mediators stimulates the activation of cytokine signaling proteins which ultimately block the interaction between the activation of insulin signaling pathway, biological function of pancreatic *β*-cells [37, 38], and conduce to insulin resistance [39]. Studies have also indicated that inflammatory cytokines such as IL-1*β*, TNF-*α*, and IL-6 which are increased in obesity and diabetes conditions modulate insulin signaling [40, 41]. Cytokines such as IL-1*β* are upregulated in pancreatic islets of patients with type 2 diabetes and regulate numerous other cytokines and chemokines, consequently increasing its production in *β* cells, engendering a vicious cycle [42–44]. A potential role of TNF-*α* has been reported in the pathogenesis of insulin resistance and type 2 diabetes [7, 45]. In the present study, the administration of nicotinamide/streptozotocin resulted in an insulin resistance and an increase in the rate of IL-1*β* and TNF-*α*, confirming their involvement in the pathogenesis of type II diabetes. Indeed, the plant extract may act as antagonist of TNF-*α* and IL-1*β* leading to the reduction of insulin resistance and to antihyperglycemia effects as observed in the study. In contrary to the study of Acharya et al. [46] where a depletion of IL-10 was observed, it was noticed in the present study, a high level of IL-10 in diabetic control group compared to normal control. In fact, IL-10 is a multiple effects cytokine acting in immunoregulation. It has been demonstrated in an in vitro study that IL-6 can cooperate with TGF-*β* to induce IL-10 production in Th17 cells [47]. *Bridelia atroviridis* was able to reduce significantly the production of IL-10 but not that of the IL-6 level. IL-6 is produced by different immune and nonimmune cells [48, 49] suggesting the implication of other factors in its synthesis. The increase in IL-6 and TNF-*α* observed in diabetes state also acts as antierythropoietic factors expressed in the present study by the decrease in erythrocytes count, hematocrit, and mean corpuscular of haemoglobin values, which are some conventional signs of anemia [36]. The proinflammatory cytokine depletion induced by the extract could justify the protective effect from anemia. The leukocytosis observed in the diabetes control is well described as diabetes complications that could result from glycation end products activation, oxidative stress, and both macrovascular and microvascular injuries caused by hyperglycemia [50]. The decrease of leukocyte counts in treated groups indicates the ability of *B. atroviridis* extract to reduce diabetes complications. The decrease in lymphocyte count observed in diabetic control group has already been reported and could be due to apoptosis induced in hyperglycemia situation [51, 52]. The administration of the plant extract for 15 days significantly improved the cells level, indicating an inhibitory effect of *B. atroviridis* on proapoptosis factors or the scavenging properties against free radical agents in lymphocytes.

Enhanced platelet reactivity has been reported in type II diabetes. In fact, hyperglycemia contributes to glycation of platelet proteins leading to altered platelet count consequently increasing their reactivity [53, 54]. However, after plant extract treatment, the level of platelet was significantly increased probably due to the improvement of the glycemia.

Reactive oxygen species (ROS) has been implicated in the mechanism of red blood cells damage [55] in the diabetic condition [56]. Previous studies reported the ROS generation in chronic high blood glucose levels, through several mechanisms including glucose autoxidation, the oxidation of protein [57, 58], or the nonenzymatic glycation of protein [59] that exacerbate oxidative stress. It is known that streptozotocin induced hydrogen peroxide (H_2_O_2_) generation and DNA fragmentation in pancreatic islets [60] which could justify the increase in catalase activity observed in the present study.

The low concentration of GSH in diabetic control suggests a depletion of this antioxidant species [61]. The nitrite rate was high in the diabetic control group probably due to streptozotocin that reacts through the nitric oxide pathway to induce *β*-cells cytotoxicity effects [62, 63]. The daily plant intake for 15 days significantly restored the catalase activity, GSH, and nitrite levels testifying the antioxidant capacity of *B. atroviridis* as seen in in vitro study. This antioxidant capacity of the extract could be assigned to its ability to scavenge free radicals or to provide a H^+^ proton or an electron to the oxidant molecules, preventing devastating effects of diabetes in treated groups [64]. Likewise, these antioxidant properties might also due to the presence of lycophene, *β*-carotene, phenolics, and flavonoids which are potent antioxidant species identified in the leaf of *B. atroviridis* [65]. In addition, corilagin, an amorphous tannin, detected in the plant extract possesses antioxidant activity [66]. Histological study of the pancreas revealed a significant reduction in the size of Langerhans islet in the diabetic control group probably due to the oxidative reaction and cytotoxic effects of streptozotocin. The size of islets was significantly increased in the treated group suggesting that *B. atroviridis* extract could regenerate pancreatic *β*-cell, thus increasing the production of insulin, and may justify the hypoglycemic effect recorded in these groups.

## 5. Conclusion

The investigation of effects of hydroethanolic extract of *Bridelia atroviridis bark* showed a decrease in hyperglycemia, inflammatory parameters, and a restoration in haematological parameters and an improvement in antioxidative status in the pancreas of nicotinamide-/streptozotocin-induced diabetes in rats. This is achieved by antidiabetic, anti-inflammatory, and antioxidant properties of the plant extract. *Bridelia atroviridis* could be a good candidate for the formulation of an antidiabetic drug plant based.

## Figures and Tables

**Table 1:** Scavenging activity or reducing power of *B. atroviridis*.

	IC_50_ (*μ*g/mL)	Positive control (*μ*g/mL)
DPPH	3.99 ± 0.35*∗*	8.93 ± 0.037
ABTS	3.23 ± 0.65	2.71 ± 0.080
NO	126.98 ± 4.61^$$$^	35.40 ± 0.049
FRAP	27.97 ± 0.25^$$^	14.05 ± 0.38

IC_50_ = inhibitory concentration 50; ^*∗*^*P* < 0.05 = more active compared to the reference; ^$$^*P* < 0.01 and ^$$$^*P* < 0.001 less active compared to the reference.

**Table 2:** Effects of hydroethanolic extract of *Bridelia atroviridis* on the variation of haematological parameters in nicotinamide-/STZ-induced diabetic rats.

Parameters	Normal control	Diabetic control	Metformin 200 mg /kg	*B. atroviridis* 50 mg/kg	*B. atroviridis* 100 mg/kg	*B. atroviridis* 200 mg/kg
RBC (106 *μ*L)	7.22 ± 0.52	4.92 ± 0.556##	8.36 ± 0.197	8.67 ± 0.182*∗∗∗*	7.82 ± 0.475*∗∗∗*	8.78 ± 0.226*∗∗∗*
HGB (g/dL)	14.10 ± 0.40	12.70 ± 0.636	14.10 ± 0.294	15.10 ± 0.799*∗*	13.80 ± 0.543	14.90 ± 0.458
HCT (%)	47.00 ± 0.40	40.50 ± 1.31##	48.40 ± 0.87*∗∗*	48.90 ± 1.60*∗∗∗*	48.40 ± 1.23*∗∗*	47.90 ± 1.29*∗∗*
MCV (fL)	71.40 ± 5.78	56.50 ± 1.56#	58.00 ± 0.45	57.00 ± 0.87	55.70 ± 1.70	54.60 ± 0.59
MCH (pg)	26.40 ± 4.37	16.30 ± 0.17##	17.00 ± 0.20	17.40 ± 0.69	16.80 ± 0.40	17.00 ± 0.22
MCHC (g/dL)	36.70 ± 3.48	29.20 ± 0.32#	29.30 ± 0.24	30.70 ± 0.65	30.10 ± 0.23	31.20 ± 0.25
WBC (103 *μ*L)	3.88 ± 0.54	6.04 ± 0.11##	7.85 ± 0.35	4.50 ± 0.26	3.84 ± 0.20*∗∗*	4.60 ± 0.40
LYMP (%)	68.10 ± 2.88	51.10 ± 2.05##	77.90 ± 2.35*∗∗∗*	74.10 ± 3.06*∗∗∗*	77.30 ± 2.02*∗∗∗*	68.40 ± 3.61*∗∗*
PLT (103 *μ*L)	870.00 ± 8.41	389.00 ± 60.60###	653.00 ± 16.10*∗∗∗*	721.00 ± 41.8*∗∗∗*	716.00 ± 13.4*∗∗∗*	617.00 ± 36.70*∗∗*

Values represent mean ± SEM (*n* = 5). #*P* < 0.05, ##*P* < 0.01, and ###*P* < 0.001: significant difference compared to normal control. *∗P* < 0.05; *∗∗P* < 0.01; and *∗∗∗P* < 0.001: significant difference related to diabetic control. Normal control = healthy animals treated with distilled water (10 mL/kg). Diabetic control = nicotinamide-/streptozotocin-induced diabetic rats treated with distilled water (10 mL/kg). Metformin 200 mg/kg = nicotinamide-/streptozotocin-induced diabetic rats treated with metformin at the dose of 200 mg/kg. *B. atroviridis* 50 mg/kg, 100 mg/kg, and 200 mg/kg = nicotinamide-/streptozotocin-induced diabetic rats treated with *B. atroviridis* extract at the doses of 50 m/kg, 100 mg/kg, and 200 mg/kg, respectively. RBC = red blood cell; HGB = haemoglobin; HCT = hematocrit; PLT = platelets; MCV = mean corpuscular volume; MCH = mean corpuscular haemoglobin; MCHC = mean corpuscular haemoglobin concentration; WBC = white blood cells; LYMP = lymphocytes; PLT = platelets.

## Data Availability

All data used to support the ﬁndings of this study are available from the corresponding author upon request.

## Abbreviations

IDF: International Diabetes Federation
IL-1*β*: Interleukin 1*β*
IL-6: Interleukin 6
TNF-*α*: Tumor necrosis factor
IL-10: Interleukin 10
ROS: Reactive oxygen species
DPPH: 1,1-diphenyl-2- picrylhydrazyl
ABTS: 2,2′-azino-bis (3-ethylbenzothiazoline-6-sulphonic) acid
FRAP: Ferric reducing antioxidant power
NO: Nitrite oxide
RSA: Radical scavenging activity
IC_50_: Inhibitory concentration 50
KITT: Insulin tolerance test index
RBC: Red blood cell
HGB: Haemoglobin
HCT: Haematocrit
PLT: Platelets
WBC: White blood cells
LYMP: Lymphocytes
PLT: Platelet.

## Supplementary Materials

Supplementary Materials The administration of the hydroethanolic extract from *Bridelia atroviridis* (Euphorbiaceae) to nicotinamide-/streptozotocin-induced diabetic rats improves blood glucose levels confirmed by the increase of *K*_iTT_ value, preventing them from anemia, leukocytopenia, and thrombocytopenia. The extract also exhibits significant regulation in the cytokine imbalance induced in diabetic conditions (Figure S1).

## References

[1] Sharma B., Balomajumder C., Roy P. (2008). Hypoglycemic and hypolipidemic effects of flavonoid rich extract from Eugenia jambolana seeds on streptozotocin induced diabetic rats. *Food and Chemical Toxicology*.

[2] Cho N. H., Shaw J. E., Karuranga S. (2018). IDF Diabetes Atlas: Global estimates of diabetes prevalence for 2017 and projections for 2045. *Diabetes Research and Clinical Practice*.

[3] Kooti W., Farokhipour M., Asadzadeh Z., Ashtary-Larky D., Asadi-Samani M. (2016). The role of medicinal plants in the treatment of diabetes: a systematic review. *Electronic Physician*.

[4] Favard P., Bonnal L., Tomen H. (2015). Protection ET risque maladie: LE CAS DU. Paludisme au cameroun. *Assurance et Gestion des Risques*.

[5] Antinori S., Galimberti L., Milazzo L., Corbellino M. (2012). Biology of human malaria plasmodia including Plasmodium knowlesi. *Mediterranean Journal of Hematology and Infectious Diseases*.

[6] Gardner M. J., Shallom S. J., Carlton J. M. (2002). Sequence of Plasmodium falciparum chromosomes 2, 10, 11 and 14. *Nature*.

[7] Donath M. Y., Dalmas É., Sauter N. S., Böni-Schnetzler M. (2013). Inflammation in obesity and diabetes: islet dysfunction and therapeutic opportunity. *Cell Metabolism*.

[8] Snow R. W., Guerra C. A., Noor A. M., Myint H. Y., Hay S. I. (2005). The global distribution of clinical episodes of Plasmodium falciparum malaria. *Nature*.

[9] Mueller I., Zimmerman P. A., Reeder J. C. (2007). Plasmodium malariae and Plasmodium ovale—the “bashful” malaria parasites. *Trends in Parasitology*.

[10] Krotoski W. A., Killick-Kendrick R., Koontz L. C. (1982). Demonstration of hypnozoites in sporozoite-transmitted plasmodium vivax infection. *The American Journal of Tropical Medicine and Hygiene*.

[11] Fernandes S. M., Cordeiro P. M., Watanabe M., Fonseca C. D. d., Vattimo M. d. F. F. (2016). The role of oxidative stress in streptozotocin-induced diabetic nephropathy in rats. *Archives of Endocrinology and Metabolism*.

[12] Chin W., Coatney G. R. (1971). Relapse activity in sporozoite-induced infections with a west african strain of plasmodium ovale. *The American Journal of Tropical Medicine and Hygiene*.

[13] Ngueyem T. A., Brusotti G., Caccialanza G., Finzi P. V. (2009). The genus Bridelia: a phytochemical and ethnopharmacological review. *Journal of Ethnopharmacology*.

[14] Adjanohoun J., Aboubakar N., Dramane K. (1996). *Contribution to Ethnobotanical and Floristic Studies in Cameroon*.

[15] Adewale O., Oloyede O. (2012). Hypoglycemic activity of aqueous extract of the bark of Bridelia ferruginea in normal and alloxan-induced diabetic rats. *Prime Research on Biotechnology*.

[16] Omeh Y. N., Onoja S. O., Ezeja M. I., Okwor P. O. (2014). Subacute antidiabetic and in vivo antioxidant effects of methanolic extract of Bridelia micrantha (Hochst Baill) leaf on alloxan-induced hyperglycaemic rats. *Journal of Complementary and Integrative Medicine*.

[17] Sokeng S., Rokeya B., Mostafa M. (2005). Antihyperglycemic effect of Bridelia ndellensis ethanol extract and fractions in streptozotocin-induced diabetic rats. *African Journal of Traditional, Complementary and Alternative Medicines*.

[18] Njamen D., Nkeh-Chungag B. N., Djiogue S. (2011). Antidiabetic properties of the methanolic extract of Bridelia grandis (Euphorbiacae) in ob/ob and db/db mice. *African Journal of Biotechnology*.

[19] Anna W. K., Shripal M. C. (2011). Antidiabetic activity of stem bark of Bridelia retusa (Linn.) Spreng. *Journal of Pharmacy Research*.

[20] Adebisi A. A., Lapido D. O., Schmelzer G. H., Gurib-Fakim A. (2008). Market survey of the barks of forest trees of phytomedicinal importance in southern Nigeria. *Plant Ressources of Tropical Africa 11(1).Medicinal Plant 1*.

[21] Odebiyi O., Sofowora E. A. (1978). Phytochemical screening of Nigerian medicinal plants II. *Lloydia*.

[22] Gul R., Jan S. U., Faridullah S., Sherani S., Jahan N. (2017). Preliminary phytochemical screening, quantitative analysis of alkaloids, and antioxidant activity of crude plant extracts from ephedra intermedia indigenous to balochistan. *The Scientific World Journal*.

[23] Aadil K. R., Barapatre A., Sahu S., Jha H., Tiwary B. N. (2014). Free radical scavenging activity and reducing power of Acacia nilotica wood lignin. *International Journal of Biological Macromolecules*.

[24] Hossain H., Ahmed T., Howlader S. I. (2012). ISSN ( print ) in-vitro antioxidant potential from the leaves of punica granatum linn. *Grown in Bangladesh*.

[25] Benzie I. F. F., Strain J. J. (1996). The ferric reducing ability of plasma (FRAP) as a measure of “antioxidant power”: the FRAP assay. *Analytical Biochemistry*.

[26] Tond S. B., Fallah S., Salemi Z., Seifi M. (2016). Influence of mulberry leaf extract on serum adiponectin, visfatin and lipid profile levels in type 2 diabetic rats. *Brazilian Archives of Biology and Technology*.

[27] Ahmed O. M., Moneim A. A., Yazid I. A., Mahmoud A. M. (2010). Antihyperglycemic, antihyperlipidemic and antioxidant effects and the probable mechanisms of action of Ruta graveolens infusion and rutin in nicotinamide-streptozotocin-induced diabetic rats. *Diabetologia Croatica*.

[28] Patarrão R. S., Lautt W. W., Macedo M. P. (2014). Assessment of methods and indexes of insulin sensitivity. Revista Portuguesa de Endocrinologia. *Diabetese Metabolismo*.

[29] Ngueguim F. T., Esse E. C., Dzeufiet P. D. D. (2015). Oxidised palm oil and sucrose induced hyperglycemia in normal rats: effects of sclerocarya birrea stem barks aqueous extract. *BMC Complementary and Alternative Medicine*.

[30] ADA (2016). Standards of medical Care in diabetes. *Diabetes Care*.

[31] Sudasinghe H. P., Peiris D. C. (2018). Hypoglycemic and hypolipidemic activity of aqueous leaf extract of Passiflora suberosa L. *Peer-Reviewed Scientific Mega Journal*.

[32] Djouwoug C. N., Gounoue R. K., Ngueguim F. T. (2021). In vitro and in vivo antiplasmodial activity of hydroethanolic bark extract of Bridelia atroviridis müll. Arg. (Euphorbiaceae) and lc-ms-based phytochemical analysis. *Journal of Ethnopharmacology*.

[33] Nandini H. S., Naik P. R. (2019). Action of corilagin on hyperglycemia, hyperlipidemia and oxidative stress in streptozotocin-induced diabetic rats. *Chemico-Biological Interactions*.

[34] Taheri Y., Suleria H. A. R., Martins N. (2020). Myricetin bioactive effects: moving from preclinical evidence to potential clinical applications. *BMC Complementary Medicine and Therapies*.

[35] Aminzadeh A., Bashiri H. (2020). Myricetin ameliorates high glucose-induced endothelial dysfunction in human umbilical vein endothelial cells. *Cell Biochemistry and Function*.

[36] Baisakhiya S., Garg P., Singh S. (2017). Anemia in patients with type II diabetes mellitus with and without diabetic retinopathy. *International Journal of Medical Science and Public Health*.

[37] Gentilini M., Duflo B. (1990). *Le Paludisme Dans Médecine Tropicale*.

[38] Good M. F., Doolan D. L. (2010). Malaria vaccine design: immunological considerations. *Immunity*.

[39] Rehman K., Akash M. S. H. (2016). Mechanisms of inflammatory responses and development of insulin resistance: how are they interlinked?. *Journal of Biomedical Science*.

[40] Xia C., Rao X., Zhong J. (2017). Role of T Lymphocytes in type 2 diabetes and diabetes-associated inflammation. *Journal of Diabetes Research*.

[41] Tong H. V., Luu N. K., Son H. A. (2017). Adiponectin and pro-inflammatory cytokines are modulated in Vietnamese patients with type 2 diabetes mellitus. *Journal of Diabetes Investigation*.

[42] Böni-Schnetzler M., Thorne J., Parnaud G. (2008). Increased interleukin (IL)-1*β* messenger ribonucleic acid expression in *β*-cells of individuals with type 2 diabetes and regulation of IL-1*β* in human islets by glucose and autostimulation. *The Journal of Clinical Endocrinology & Metabolism*.

[43] Pages F., Orlandipradines E., Corbel V. (2007). Vecteurs du paludisme: biologie, diversité, contrôle et protection individuelle. *Médecine et Maladies Infectieuses*.

[44] Ehses J. A., Lacraz G., Giroix M.-H. (2009). IL-1 antagonism reduces hyperglycemia and tissue inflammation in the type 2 diabetic GK rat. *Proceedings of the National Academy of Sciences*.

[45] Rodríguez M., Pérez L., Caicedo J. C. (2009). Composition and biting activity ofAnopheles (Diptera: Culicidae) in the amazon region of Colombia. *Journal of Medical Entomology*.

[46] Acharya A., Thakur S., Muddapur M. (2015). Effect of scaling and root planing on serum interleukin-10 levels and glycemic control in chronic periodontitis and type 2 diabetes mellitus. *Journal of Indian Society of Periodontology*.

[47] Jin J.-O., Han X., Yu Q. (2013). Interleukin-6 induces the generation of IL-10-producing Tr1 cells and suppresses autoimmune tissue inflammation. *Journal of Autoimmunity*.

[48] Garg R. (2000). Cerebral malaria. *The Journal of the Association of Physicians of India*.

[49] Kristiansen O. P., Mandrup-Poulsen T. (2005). Interleukin-6 and diabetes: the good, the bad, or the indifferent?. *Diabetes*.

[50] Moradi S., Jafarian-Kerman S. R., Salari F., Rohani F. (2012). Association between diabetes complications and leukocyte counts in Iranian patients. *Journal of Inflammation Research*.

[51] Otton R., Soriano F., Verlengia R., Curi R. (2004). Diabetes induces apoptosis in lymphocytes. *Journal of Endocrinology*.

[52] Arya A., Garg S., Kumar S., Meena L., Tripathi K. (2013). Estimation of lymphocyte apoptosis in patients with chronic, non healing diabetic foot ulcer. *International Journal of Medical Science and Public Health*.

[53] Vinik A. I., Erbas T., Park T. S., Nolan R., Pittenger G. L. (2001). Platelet dysfunction in type 2 diabetes. *Diabetes Care*.

[54] Shilpi K., Potekar R. M. (2018). A study of platelet indices in type 2 diabetes mellitus patients. *Indian Journal of Hematology and Blood Transfusion*.

[55] Rao G., Kamath U., Raghothama C., Pradeep K. S., Rao P. (2003). Maternal and fetal indicators of oxidative stress in various obstetric complications. *Indian Journal of Clinical Biochemistry*.

[56] Singh D. K., Winocour P., Farrington K. (2009). Erythropoietic stress and anemia in diabetes mellitus. *Nature Reviews Endocrinology*.

[57] Pérignon J. L., Druilhe P. (1994). Immune mechanisms underlying the premunition against Plasmodium falciparum malaria. *Memórias Do Instituto Oswaldo Cruz*.

[58] Maritim A. C., Sanders R. A., Watkins J. B. (2003). Diabetes, oxidative stress, and antioxidants: a review. *Journal of Biochemical and Molecular Toxicology*.

[59] Obi R., Okangba C., Nwanebu F., Ndubuisi U., Orji N. (2010). Premunition in Plasmodium falciparum malaria. *African Journal of Biotechnology*.

[60] Aronoff S. L., Berkowitz K., Shreiner B., Want L. (2004). Glucose metabolism and regulation: beyond insulin and glucagon. *Diabetes Spectrum*.

[61] Broberger C. (2005). Brain regulation of food intake and appetite: molecules and networks. *Journal of Internal Medicine*.

[62] Szkudelski T. (2012). Streptozotocin-nicotinamide-induced diabetes in the rat. Characteristics of the experimental model. *Experimental Biology and Medicine*.

[63] Steiner D. F., Oyer P. E. (1967). The biosynthesis of insulin and a probable precursor of insulin by a human islet cell adenoma. *Proceedings of the National Academy of Sciences*.

[64] Moukette B. M., Ama Moor V. J., Biapa Nya C. P. (2017). Antioxidant and synergistic antidiabetic activities of a three-plant preparation used in Cameroon folk medicine. *International Scholarly Research Notices*.

[65] Sokefun O. O., Eleyowo O. O. (2017). In vitro Antioxidant and Antibacterial activity of Bridelia atroviridis. *Arasado*.

[66] Yakubu O. F., Adebayo A. H., Iweala E. E., Adelani I. B., Ishola T. A., Zhang Y.-J. (2019). Anti-inflammatory and antioxidant activities of fractions and compound from Ricinodendron heudelotii (Baill.). *Heliyon*.

