# Supplementary material for: Hydroethanolic Extract from Bridelia atroviridis Müll. Arg. Bark Improves Haematological and Biochemical Parameters in Nicotinamide-/Streptozotocin-Induced Diabetic Rats
Source: Evid Based Complement Alternat Med. 2020 Nov 25;2020:3160834. doi: 10.1155/2020/3160834 (PMC7714572; doi:10.1155/2020/3160834)
Supplement: Supplementary Materials — The administration of the hydroethanolic extract from Bridelia atroviridis (Euphorbiaceae) to nicotinamide-/streptozotocin-induced diabetic rats improves blood glucose levels confirmed by the increase of KiTT value, preventing them from anemia, leukocytopenia, and thrombocytopenia. The extract also exhibits significant regulation in the cytokine imbalance induced in diabetic conditions (Figure S1). [file 3160834.f1.pdf]

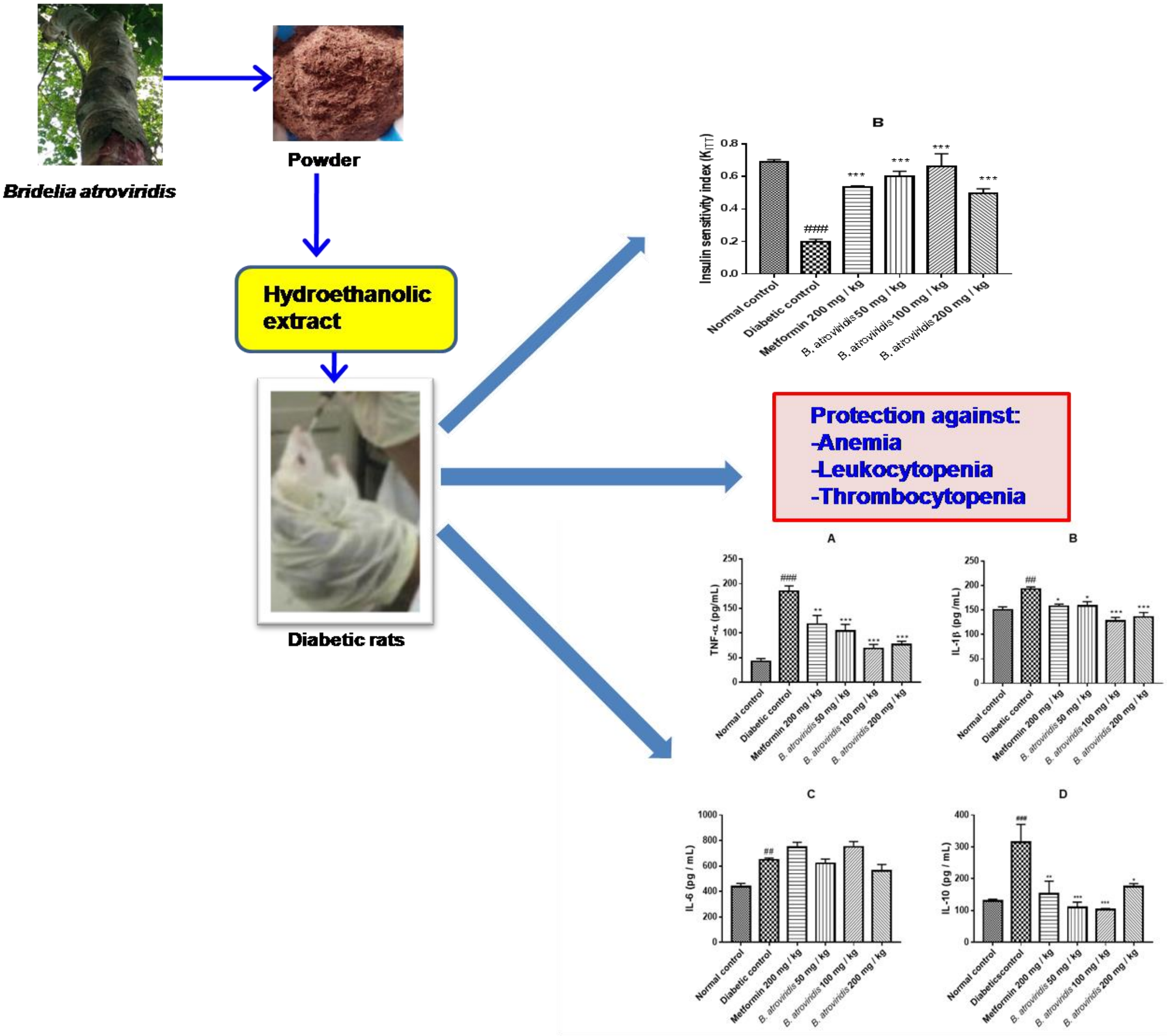

Fig S1: Effects of hydro-ethanolic extract from Bridelia atroviridis bark on haematological and biochemical parameters in nicotinamide/streptozotocin-induced diabetic rats.

The administration of the hydro-ethanolic extract from Bridelia atroviridis (Euphorbiaceae) to nicotinamide/streptozotocin-induced diabetic rats improves blood glucose levels confirmed by the increase of KiTT value; prevents from anemia, leukocytopenia and thrombocytopenia. The extract also exhibits significant regulation in the cytokines imbalance induced in diabetic conditions.
